# Was ist neu … bei den Faktor-XI-Inhibitoren: DOAK 2.0?

**DOI:** 10.1007/s00101-024-01396-3

**Published:** 2024-04-03

**Authors:** A. Schlake, P. Scheiermann, C. F. Weber

**Affiliations:** 1 Abteilung für Anästhesiologie, Intensiv- und Notfallmedizin, Asklepios Klinik Wandsbek, Alphonsstraße 14, 22043 Hamburg, Deutschland; 2 Klinik für Anaesthesiologie, LMU Klinikum München, München, Deutschland; 3 Klinik für Anästhesiologie, Intensivmedizin und Schmerztherapie, Universitätsklinikum Frankfurt, Frankfurt, Deutschland

## Einführung

In den vergangenen Jahren haben sich die direkten oralen Antikoagulanzien (DOAK) bei den meisten Indikationen als Standardtherapeutika für Patienten mit Antikoagulationsbedarf durchgesetzt. Sie gelten als besser steuerbar, effektiver und sicherer als Vitamin-K-Antagonisten. Unter anderem, weil die DOAK-Einnahme weiterhin mit erhöhtem Blutungsrisiko assoziiert ist und in absehbarer Zeit der Patentschutz für die etablierten DOAK ausläuft, wird seit Längerem an der Entwicklung von Nachfolgesubstanzen geforscht. Faktor-XI-Inhibitoren könnten eine Alternative sein.

## Das ist neu!

**Figure 1:**
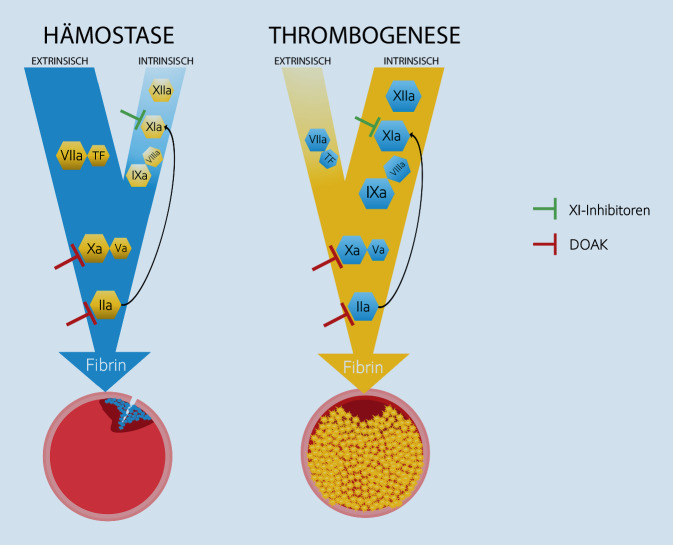

Aus hämostaseologischer Sicht ist die Inhibition des Gerinnungsfaktors XI ein vielversprechender Ansatz für neu zu entwickelnde Antikoagulanzien, denn Faktor XI ist Bestandteil der intrinsischen Gerinnungskaskade und vermittelt nach Aktivierung durch Faktor XII (Kontaktaktivierung) die intraluminale *Thrombogenese *(Abb. 1). Faktor XI spielt hingegen in der gewebsfaktorvermittelten *Hämostase* (extrinsischer Weg) nur eine untergeordnete Rolle, die sich auf Konsolidierung existierender Gerinnsel und die Verstärkung ihrer Resistenz gegenüber der Fibrinolyse beschränkt [1]. Dass eine Inhibition von Faktor XI einerseits die Thrombogenese signifikant beschränken und andererseits aber die Hämostase nahezu unbeeinflusst lassen würde, lassen auch epidemiologische Studien an Patienten mit hereditärem Faktor-XI-Mangel (Hämophilie C) vermuten, die überdurchschnittlich selten an venösen Thromboembolien oder ischämischem Schlaganfall erkranken [2], ohne bei chirurgischen Eingriffen oder im Zusammenhang mit Traumata eine vermehrte Blutungsneigung aufzuweisen.

**Figure 2:**
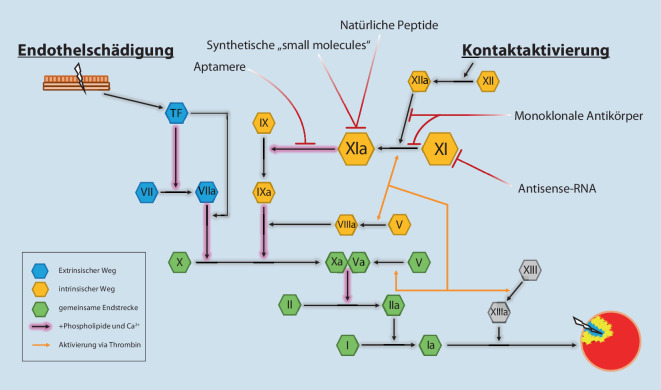

Für die pharmakologische Hemmung des Faktor XI gibt es eine Vielzahl möglicher Ansätze (Abb. 2; [3]). Neben Syntheseunterbrechung durch Antisense-RNA oder Antikörper gegen XI bzw. seine aktive Form XIa, welche sich aufgrund des langen Wirkeintritts (Antisense-RNA) oder langer Halbwertszeit (Antikörper) und der damit verbundenen schwierigeren Steuerung vermutlich nicht durchsetzen werden können, stehen natürliche Peptide, Aptamere und synthetische „small molecules“ im Fokus. Natürliche Peptide hemmen die Aktivierung von Faktor XI; Aptamere (Einzelstrangoligonukleotide mit hoher Affinität an Zielstrukturen auf Proteinen/Nukleinsäuren) sind zusätzlich auch in der Lage, Faktor XIa zu inhibieren. Beide Wirkstoffgruppen zeigen einen schnellen Wirkeintritt – sind aber noch nicht in klinischen Studien an Menschen untersucht worden.

Die Gruppe der „small molecules“ ist mit Blick auf allgemeine Charakteristika wie Interaktion mit anderen Wirkstoffen, Steuerbarkeit, Sicherheit und Halbwertszeit aus aktueller Sicht am vielversprechendsten. Mehrere Phase-2-Studien [4, 5] und systematische Metaanalysen [6] hatten erfolgversprechende Ergebnisse erzielt. Im November 2023 musste jedoch die Fa. Bayer mit dem vorzeitigen Abbruch der OCEANIC-AF-Phase-3-Studie einen großen Rückschlag hinnehmen: Der Wirkstoff Asundexian zeigte sich in einer Interimsanalyse im Vergleich mit dem Kontrollarm (Apixaban) bei Patienten mit Vorhofflimmern unterlegen.

Das Marktpotenzial für Antikoagulanzien ist sehr groß – in Deutschland erhalten mehr als 10 Mio. Patienten orale Antikoagulanzien; für die Hersteller ein Milliardenmarkt [7]. Weil der Patentschutz der gegenwärtig häufig verschriebenen DOAK ab 2026 abläuft, ist die Entwicklung von Nachfolgesubstanzen ein hoch priorisiertes Ziel. Ob sich die Faktor-XI-Inhibitoren durchsetzen, und wenn ja – aus welcher Subgruppe – ist aktuell noch völlig unklar. Unklarheit herrscht auch beim Monitoring dieser Substanzen, denn die erwartbare isolierte Verlängerung der aPTT bei Einnahme von Faktor-XI-Inhibitoren [1] stünde wohl in keinem Verhältnis zur antikoagulatorischen Wirkung der Substanz – die leidliche Auseinandersetzung mit diesem Dilemma sind wir Anästhesist:Innen schon von den klassischen DOAK gewohnt.

## Fazit für die Praxis

Die Hemmung von Gerinnungsfaktor XI ist ein vielversprechender Ansatz bei der Entwicklung von Nachfolgesubstanzen für die etablierten DOAK. Dem Konzept nach würde eine Hemmung von Faktor XI die intrinsisch vermittelte Thrombogenese einschränken, ohne die extrinsisch vermittelte Hämostase zu limitieren, weil die gemeinsame Endstrecke über Faktor X und Thrombin nicht eingeschränkt wäre. Zum gegenwärtigen Zeitpunkt sind verschiedene Substanzen in unterschiedlichen Entwicklungsstadien auf dem Niveau von Phase-2- und Phase-3-Studien.
